# Osteopetrotic (op/op) mice have reduced microglia, no Aβ deposition, and no changes in dopaminergic neurons

**DOI:** 10.1186/1742-2094-4-31

**Published:** 2007-12-20

**Authors:** Yoichi Kondo, Cynthia A Lemere, Timothy J Seabrook

**Affiliations:** 1 Department of Medical Sciences, School of Veterinary Medicine, University of Wisconsin, Madison, WI 53706, USA; 2 Center for Neurologic Diseases, Brigham and Women's Hospital, Harvard Medical School, Boston, MA 02115, USA

## Abstract

**Background:**

Activation of microglia is a part of the inflammatory response in neurodegenerative diseases but its role in the pathophysiology of these diseases is still unclear. The osteopetrotic (op/op) mouse lacks colony-stimulating factor-1 (CSF-1) and thus has a deficiency in microglia and macrophages. Prior reports have demonstrated that op/op mice deposit amyloid β (Aβ) plaques, similar to those found in Alzheimer's disease. The purpose of these studies was to confirm this and to determine if the lack of CSF-1 affects the development of dopaminergic neurons and the expression of CD200, a known microglial inhibitory protein.

**Method:**

We examined the central nervous system of op/op mice at 30 days, 60 days and 7 months of age and wildtype littermates at 30 days using immunohistochemistry and histochemistry.

**Results:**

We found a decrease in the number of microglia in 1 month-old op/op mice compared to wildtype (WT) littermates as measured by CD11b, CD45, CD32/16, CD68, CD204 and F4/80 immunoreactivity. Aβ plaques were not detected, while the number of dopaminergic neurons appeared normal. The expression of CD200 appeared to be normal, but there appeared to be a lower expression in the substantia nigra.

**Conclusion:**

In contrast to a prior report we did not detect Aβ deposition in the central nervous system of op/op mice at 30 days, 60 days or 7 months of age and there was a normal number of dopaminergic neurons. This indicates that op/op mice may be useful to examine the effects of microglia on neurodegenerative disease progression by breeding them to different transgenic mouse models. In addition, the lack of CSF-1 does not appear to affect CD200 expression by neurons but we did note a decrease in the substantia nigra of op/op and WT mice, suggesting that this may be a mechanism by which microglia control may be attenuated in this specific area during Parkinson's disease.

## 1. Introduction

Alzheimer's disease (AD) and Parkinson's disease (PD) affect an increasing number of people as the population ages [1]. In AD brain, activated microglia are localized to areas of β amyloid (Aβ) plaque [1,1-3], whilst in PD they are found in the substantia nigra (SN), the site of dopaminergic cell loss in PD [4,5]. In both AD and PD there is epidemiological evidence [6,7] and animal model data [8,9] to suggest that suppressing inflammation is beneficial. However, microglia activation can reduce Aβ plaque development in AD mouse models [8,10,11] and activated microglia secrete neurotrophic factors [8,12]. Thus the role of microglia during neurodegeneration is complex. Alternative animal models may assist in addressing these difficult remaining questions.

Osteopetrotic (*Csf1*^*op*^*/Csf1*^*op *^or commonly called op/op) mutant mice are deficient in colony stimulating factor-1 (CSF-1) due to a naturally occurring inactivating mutation [13,14]. CSF-1, also known as macrophage colony stimulating factor, has an important role in the proliferation and differentiation of macrophages [15], subsequently op/op mice are reported to have a deficiency in tissue macrophages, including microglia [16]. However, this deficiency may not be absolute, and the number of macrophages appears to increase as the mice age [17]. If the number of microglia is reduced op/op mice may be an appropriate model to investigate the role of microglia and astrocytes in neurodegenerative disease.

Microglia are the main immune type cell of the central nervous system (CNS) and therefore are crucial to the defense of the brain against invading micro-organisms, tumors and following trauma. However, uncontrolled activation may have deleterious outcomes. The mechanisms by which the CNS accomplishes this balancing act are slowly being elucidated but are known to include astrocytes, neurons and microglia themselves. Some of the mechanisms include the production of soluble factors such as neurotrophins, cytokines and ligands such as CD200 and its receptor CD200R and CD22 plus its receptor CD45 [18,19]. It is unknown if the absence of microglia will affect the appearance of these downregulatory mechanisms.

The purpose of these experiments was to determine the presence of microglia/macrophages in the CNS of op/op mice using a panel of antibodies and if the reduced density of microglia affected the appearance of the downregulatory protein CD200 on neurons. In addition, we examined whether the lack of CSF-1 and microglia resulted in the deposition Aβ plaques or dopaminergic cell loss.

## 2. Experimental procedure

### 2.1 Mice

Breeding pairs of *Csf1*^+/op ^mice (B6C3Fe genetic background) were purchased from the Jackson Laboratory (Bar Harbor, ME) and bred at the School of Veterinary Medicine, University of Wisconsin-Madison. Homozygous op/op mice, identified by the lack of incisors around postnatal day 12, were fed with powdered rodent chow (Harlan, Indianapolis, IN) and liquid rodent diet (Research Diets Inc., New Brunswick, NJ) after weaning. The heterozygous or WT littermates, as identified by the normal appearance of teeth, were used as controls. All animal procedures were approved by the University's Animal Care and Use Committee.

### 2.2 Tissue collection

At 30 days, 60 days and 7 months of age op/op mice and 30 day-old WT littermates (n = 3, each group) were deeply anaesthetized and perfused with saline followed by 4% paraformaldehyde in 0.1 M phosphate buffer (pH 7.4). The brain was removed and placed in 4% paraformaldehyde for 8–10 hours, sucrose protected, embedded in OCT (Sakura, Tokyo, Japan), and frozen in a dry ice cooled isopentane bath. Tissue was then stored at -80°C until use.

### 2.3 Immunohistochemistry, histochemistry and image analysis

Eight micron sections were cut using a cryostat, mounted on glass slides and immunohistochemistry performed using the ELITE ABC kit (Vector Laboratories, Burlingame, CA) and diaminobenzidine as previously described [20]. The dilution and source of the utilized antibodies are shown in Table 1.

**Table 1:** Antibodies used for immunohistochemistry

Antigen (catalogue number)	Antibody type	Dilution	Source
CD16/CD32 (553140)	Rat monoclonal	1:2000	BD PharMingen (San Jose, CA)
CD11b (MCA711)	Rat monoclonal	1:250	Serotec (Raleigh, NC)
CD45 (MCA 1031G)	Rat monoclonal	1:5000	Serotec
CD68 (MCA 1957)	Rat monoclonal	1:500	Serotec
CD204 (MCA 1322)	Rat monoclonal	1:1000	Serotec
F4/80 (MCA497R)	Rat monoclonal	1:250	Serotec
GFAP (Z 0334)	Rabbit polyclonal	1:500	Dakocytomation (Carpinteria, CA)
DW14 (anti-Aβ) none	Rabbit polyclonal	1:1000	Dr. Selkoe (Boston, MA)
R1282 (anti-Aβ) none	Rabbit polyclonal	1:1000	Dr. Selkoe
Aβ 40 (44–348)	Rabbit polyclonal	1:250	Biosource (Camarillo, CA)
Aβ 42 (44–344)	Rabbit polyclonal	1:250	Biosource
tyrosine hydroxylase (AB152)	Rabbit polyclonal	1:1000	Chemicon (Temecula, CA)
CD200 (552512)	Rat monoclonal	1:250	BD PharMingen
MHC II (553621)	rat monoclonal	1:500	BD PharMingen

Fibrillar Aβ was detected using 1% aqueous Thioflavin S followed by differentiation in 3 changes of 80% ethanol and examination by fluorescent microscopy. Congo red staining was performed by immersing slides in a solution of 0.1% Congo red solution for 5 minutes and differentiation in a solution of 85% ethanol and 0.2% potassium hydroxide. Slides were then examined using polarizing microscopy and light microscopy.

Sections of spleen and brain from plaque-bearing 12 month-old J20 amyloid precursor protein (APP)-tg mice [21] were included in all histochemistry and immunohistochemistry experiments as positive controls. Negative controls included sections incubated with normal IgG from the same species as the primary antibody.

To quantify CD11b immunoreactivity, acquisition of images was performed in a single session using a SPOT camera (Sterling Heights, MI) using 3 equidistant (~100 μm) tissue sections. Computer assisted image analysis was performed on images of hippocampus and SN using IP Lab Spectrum 3.1 Image Analyzer (Fairfax, VA). The threshold of detection was held constant during analysis. Percent area occupied by CD11b immunoreactivity was calculated for hippocampus and SN separately.

### 2.4 Data analysis

Using Prism software (GraphPad Software Inc., San Diego, CA), statistical analysis was determined by the Mann Whitney U test with a p value of < 0.05 considered significant. All values reported include the average ± SEM.

## 3. Results

### 3.1 Microglia are significantly reduced in 30 day old op/op mice

**Figure 1:**
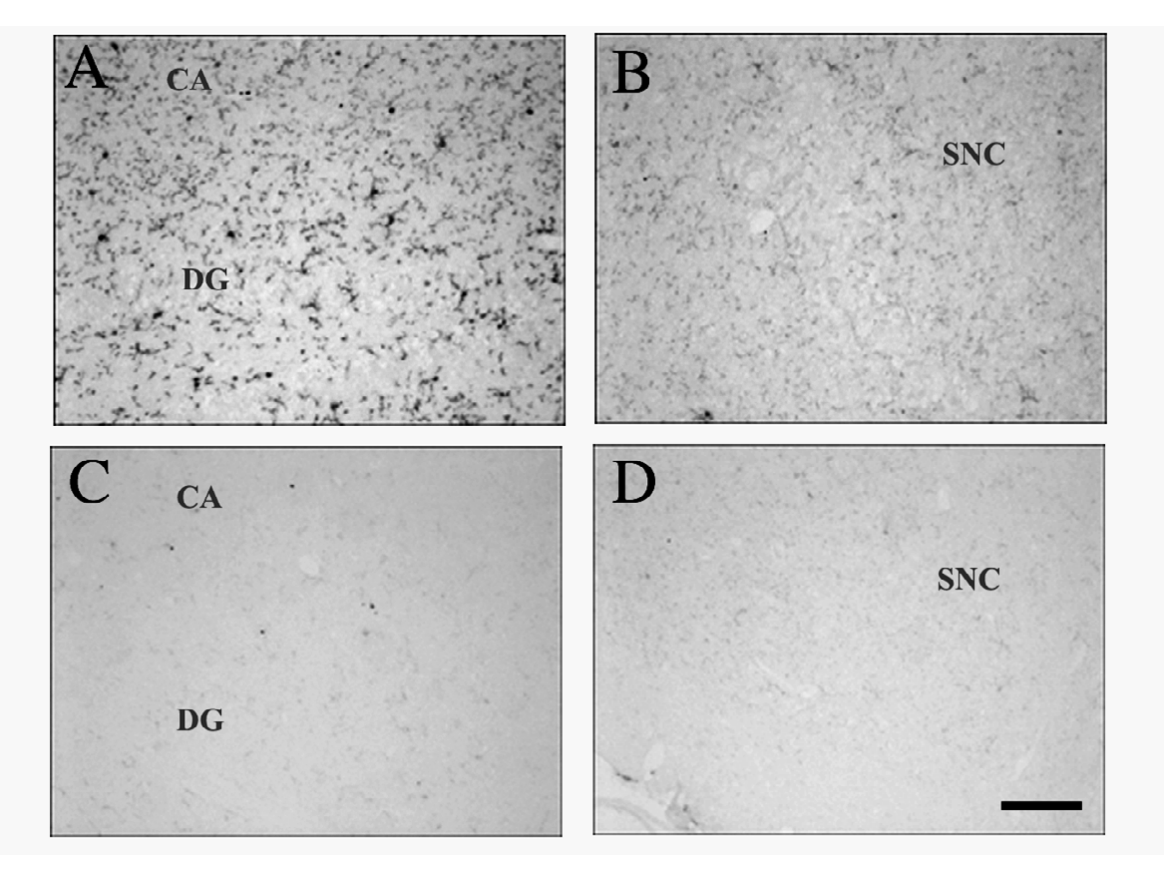
**Expression of CD11b in the hippocampus and substantia nigra of 30-day-old op/op and WT mice**. Photomicrographs show that WT mice expressed CD11b in the hippocampus (A) with lower amounts detected in the substantia nigra (B). CD11b immunoreactivity was lower in op/op mice in both the hippocampus (C) and substantia nigra (D) when compared to WT littermates. Scale bar – 100 μm.

**Figure 2:**
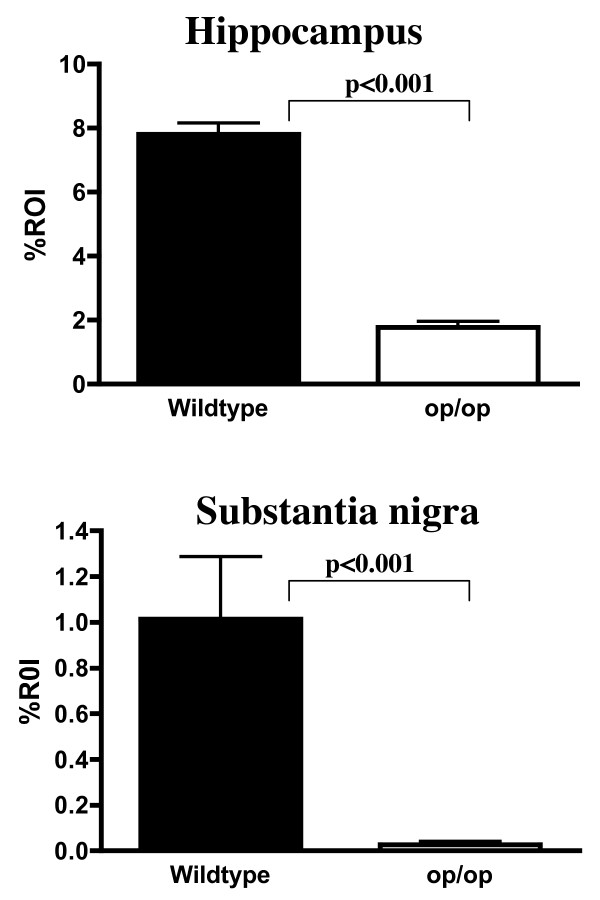
**Quantification of CD11b expression in the hippocampus and substantia nigra**. Computer assisted quantification of CD11b immunoreactivity demonstrated a significant difference in the hippocampus between WT (n = 3) and op/op (n = 3) mice at 30 days of age. This was also seen in the substantia nigra. Note the lower values in the substantia nigra compared to the hippocampus in both WT and op/op mice. All values are expressed as mean ± SEM and statistical significance was determined using the Mann Whitney U non-parametric test of significance. ROI – region of interest.

**Figure 3:**
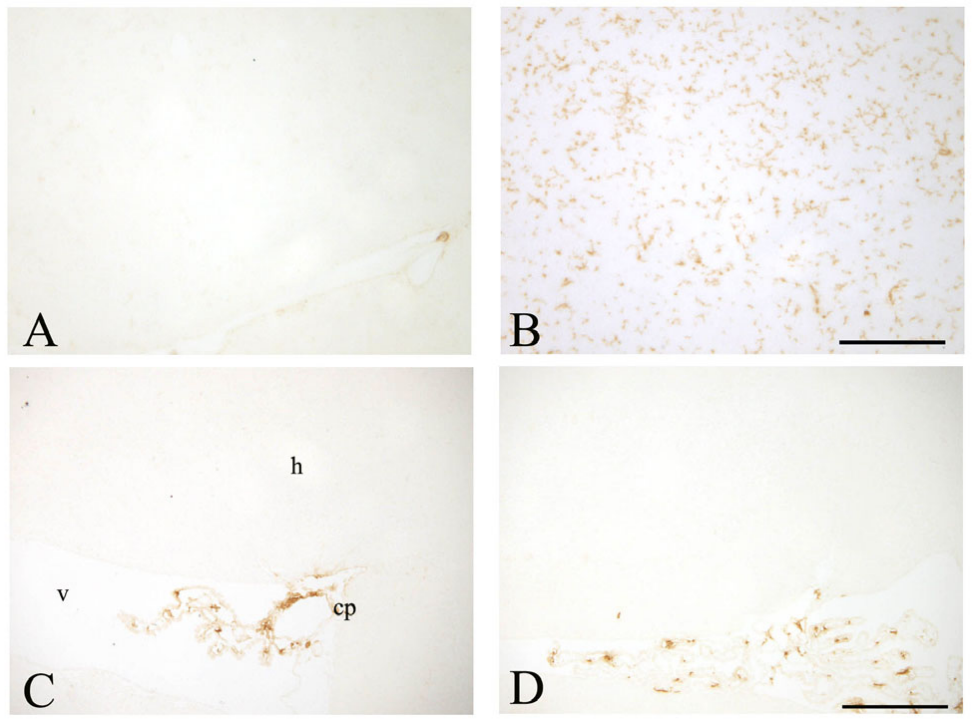
**CD45 and MHC II immunoreactivity in op/op and wildtype mice**. CD45 immunoreactivity is reduced in the hippocampus of op/op mice (A) as compared to age matched wildtype mice (B). Only larger round cells located near blood vessels were labeled in the op/op mice, whilst in wildtype mice ramified microglia can be found. In both op/op (C) and wildtype (D) mice MHC II immunoreactive cells were found solely in the choroid plexus, while the overlying hippocampus was devoid of labeled cells. scale bar – 100 μm.

Immunohistochemistry demonstrated a significant decrease in CD11b expression in the hippocampus and SN of 30 day-old op/op mice compared to WT littermates (Figure 1 and 2). The majority of the residual CD11b-expressing cells in the op/op CNS were ramified and therefore can be tentatively identified as microglia. Immunostaining with CD45 confirmed the reduced number of microglia within the brain of op/op mice (Figure 3). Note that the CD45 antibody labeled fewer cells in the hippocampus of wildtype mice as compared to the CD11b antibody. Additionally CD32/16 (FcγIII/II), CD68 and F4/80 were all reduced in the CNS of the 30 day-old op/op mice compared to WT littermates. We also examined the brains for MHC II expression and found that both op/op and WT mice expressed this marker only in cells located in the choroid plexus (Figure 3). Based on their morphology and position, the MCH II+ cells are likely macrophages in the stroma of the choroid plexus. Therefore, it appears that not all cells of the macrophage lineage are absent from the brain.

There appeared to be an increase in the number of CD11b and CD45 positive cells in the brains of op/op mice at 7 months of age, however WT littermates were not available to perform a quantitative comparison.

Normal numbers of astrocyte, as detected using an anti-GFAP antibody were found in op/op mice (data not shown).

### 3.2 CD200 expression in op/op mice

**Figure 4:**
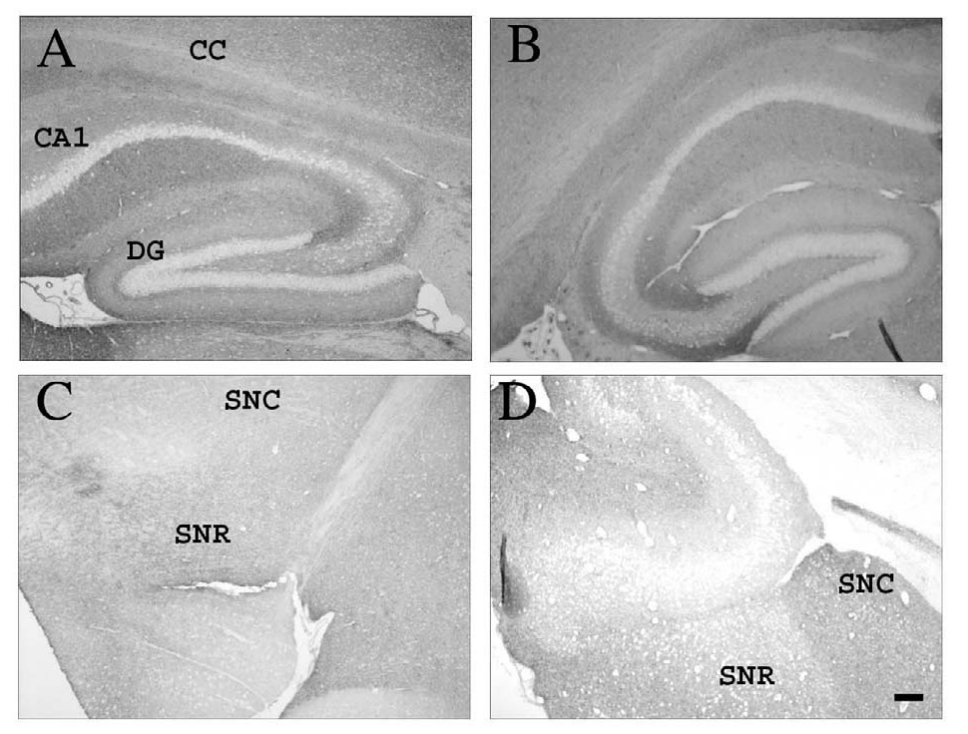
**CD200 immunoreactivity is similar between WT and op/op mice**. Photomicrographs demonstrate CD200 expression in the hippocampus of both WT (A) and op/op mice (B) at 30 days of age. There is wide spread staining found throughout the neuropil of the hippocampus except the CA regions and dentate gyrus. There was less CD200 immunoreactivity in the substantia nigra of both WT (C) and op/op mice (D). CA1- field CA1 of hippocampus, CC- corpus callosum, DG- dentate gyrus, SNR- substantia nigra, reticular, SNC- substantia nigra, compact Scale bar – 100 μm.

CD200 is a widely expressed membrane protein containing 2 Ig superfamily domains, with its receptor expressed on myeloid cells including microglia [22]. Using immunohistochemistry we found no difference in the expression of CD200 in the hippocampus or SN of op/op mice when compared to WT mice (Figure 4). As previously reported CD200 protein was found on neuronal membranes throughout the CNS, with some exceptions including the neurons in the dentate gyrus and CA regions of the hippocampus (Figure 4). Interestingly, there appeared to be less neuronal expression of CD200 in the SN compared to the hippocampus for both lines of mice.

### 3.3 Aβ plaques and dopaminergic neurons

**Figure 5:**
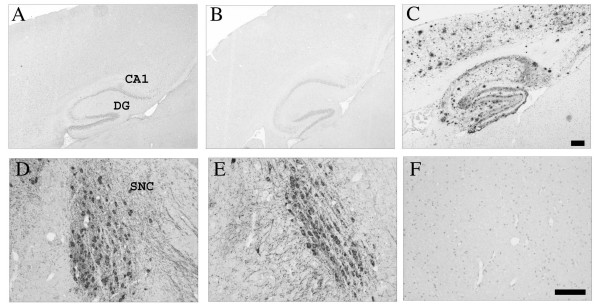
**Aβ plaques were not detected but tyrosine hydroxylase positive neurons were present in op/op mice**. The rabbit polyclonal anti-Aβ antibody, DW14, did not detect Aβ deposition in WT (A) or op/op mice (B) in the hippocampus or overlying cortex of 30 day-old mice. A cryosection from a 12 month-old APP-tg mouse (C) was included in the same immunohistochemistry experiment as a positive control and shows abundant Aβ plaques. Tyrosine hydroxylase (TH) was used to immunolabel dopaminergic neurons in the substantia nigra. There were comparable numbers of TH expressing neurons in WT (D) and op/op (E) mice. The morphology of the dopaminergic neurons was similar in both groups of 30 day-old mice. An adjacent section was incubated with normal rabbit IgG as a negative control (F) for the TH immunostaining and shows no labeled cells. CA1- field CA1 of hippocampus, CC- corpus callosum, DG- dentate gyrus, SNC- substantia nigra, compact. Scale bar A-C – 250 μm Scale bar D-F – 100 μm.

**Figure 6:**
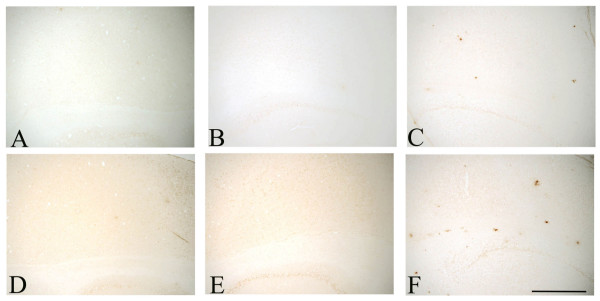
Cryosections from 30 day old op/op, wildtype and 12 month old APP-tg mice were examined using commercially available polyclonal antibodies for Aβ40 and Aβ42. There was no Aβ40 immunoreactivity in op/op (A), wildtype (B) mice, whilst the APP-tg (C) mouse section showed some dense plaques. The Aβ42 specific antibody demonstrated a similar finding with op/op (D) and wildtype (E) mice having no plaques, whilst APP-tg (F) mice showed clear labeling of plaques. scale bar – 500 μm.

We examined the CNS of 30 day, 60 day and 7 month-old op/op mice and WT littermates for the presence of Aβ plaques. Using 2 different polyclonal Aβ antibodies, R1282 and DW14 [20] we did not detect Aβ deposition in the CNS of either op/op or WT mice (Figure 5). Commercially available polyclonal antibodies specific for Aβ40 and Aβ42 did not label plaques in op/op mice or wildtype mice (Figure 6). In all cases we could detect Aβ in the CNS of an APP transgenic mouse brain in the same staining run and none was found when normal IgG was included as a negative control. To confirm the immunohistochemical data we also performed Thioflavin S and Congo red histochemistry, both of which were negative in op/op and WT mice. Therefore, based on this evidence we conclude that Aβ is not deposited in op/op or WT mice.

To examine dopaminergic neurons we performed immunohistochemistry using an antibody specific for tyrosine hydroxylase (TH). Normal numbers and morphology of TH positive neurons were found in the SN of both op/op and WT mice at 30 days, 60 days and 7 months of age (Figure 4).

## 4. Discussion

The role of microglia and astrocytes in the inflammatory cascade during neurodegenerative diseases is becoming better delineated and may be a therapeutic target. However, the exact contribution of each glia cell type is difficult to determine in vivo due to the extensive communication between astrocytes and microglia. In addition, both types of glia can produce an overlapping array of cytokines, chemokines and other molecules during an inflammatory response [2]. It has been reported that op/op mice have reduced numbers of microglia [16], thus these mice may allow us to examine whether reduced numbers of microglia affect the progression of neurodegenerative diseases following crossbreeding to AD mouse models or pharmacological induced disease, such as 1-methyl-4-1,2,3,6-tetrahydropyridine (MPTP). However, it has been unclear if op/op mice have normal dopaminergic neuronal development or if they deposit Aβ plaques under normal conditions. In addition, the activation status of microglia in op/op mice is still unclear. Without this basic data, the suitability of op/op mice as a model in neurodegenerative diseases was unknown, for example if there are deficiencies in dopaminergic neurons, op/op mice would not make a good model for PD.

There is evidence to suggest that the number of microglia in op/op mice may not be decreased compared to WT mice [23,24], whilst other reports demonstrate a significant reduction in microglia [16,25]. Our data supports the latter reports, as 30 day-old op/op mice had significantly reduced numbers of CD11b positive cells in both the hippocampus and SN. In addition, a panel of microglia/macrophage markers including FcγIII/II receptors (CD16/CD32), CD68, CD45 and the scavenger receptor class A (CD204), immunolabeled fewer cells in 30 day-old op/op mice compared to age-matched WT littermates. This suggests that the residual microglia in op/op mice are not activated or expressing alternative membrane markers compared to WT mice. There did appear to be population of macrophages like cells expressing MHCII in the choroid plexus of both op/op and wildtype mice. In both WT littermates and op/op mice there were fewer microglia, as measured by CD11b immunoreactivity, in the SN compared to the hippocampus. This is in direct contrast to a previous report demonstrating that the density of microglia is greater in SN compared to hippocampus [26], though there are numerous differences between this report and the previous. Among these differences are the age of the mice examined, antibodies used and the strain of mice. Further studies are required to determine which of these variables is responsible for the difference. However, from the present study we can conclude that microglia are indeed reduced in 30 day old op/op mice.

There are extensive interconnections between neurons, microglia and astrocytes to control the activation and function of microglia [18]. Some of the mechanisms by which neurons downregulate microglia include the production of neurotrophins [27], CD200 [28] and CD22 [29]. Microglia express both CD45, the receptor for CD22 and the CD200 receptor. Absence of neuronal CD200 leads to an accelerated microglia response in both the facial nerve transection model and experimental autoimmune encephalitis [28]. There was no difference in the neuronal expression pattern of CD200 when op/op and WT mice were compared, therefore it appears that the lower number of microglia in op/op mice does not affect the development of this key downregulatory glycoprotein. It would be interesting to determine if this also applies to other known downregulatory proteins and mechanisms. However, we did note that CD200 immunoreactivity appeared to be less intense in the SN compared to the hippocampus. This may be one mechanism by which the SN is more susceptible to LPS induced damage compared to other CNS regions [30], as the local neurons may not be able to control the activation of the microglia. This may have implications for Parkinson's disease and other neurodegenerative diseases. Further studies to better quantify CD200 protein in discrete brain regions will be informative. Interestingly a recent report has demonstrated that CD200 is decreased in the hippocampus of older rats suggesting that this may be a mechanism by which microglia activation control is not as tightly regulated as rats age [31]. However, our current study demonstrates that the absence of microglia and CSF-1 does not affect neuronal expression of CD200.

Previous reports have demonstrated that 30 day-old op/op mice deposit Aβ plaques in several regions of the CNS, including the cerebral cortex, hippocampus and hypothalmus [32,33]. However, in the current study we did not detect Aβ plaques using four different Aβ antibodies (both polyclonal and monoclonal antibodies), Congo red or Thioflavin S histochemistry in 30 day, 60 day and 7 month old op/op mice. A possibility for the discrepancy may be a non-specific binding of the Aβ antibody used in the previous study. The previous study used only a single antibody, did not report the dilution used and did not describe the results of appropriate positive and negative controls. Therefore, based on our extensive and careful examination of the brain of op/op mice we conclude that op/op mice do not deposit Aβ under normal conditions more so than WT mice.

There are a normal number of TH positive neurons, with the appropriate morphology seen in 30 day-old op/op mice as compared to WT littermates. It has been demonstrated that an injection of LPS into the SN causes a loss of TH expressing neurons, thus it is likely that op/op mice with the lower number of microglia would be resistant to this phenomenon. It has been demonstrated that mice with lower numbers of microglia and increased numbers of astrocytes in the SN are resistant to MPTP induced dopaminergic neuron loss [34]. If this is the case then it would be expected that op/op mice would be highly resistant to dopaminergic cell loss following MPTP treatment. The use of op/op mice would allow the in vivo study of astrocytes in mouse models of PD without the confounding presence of microglia.

## 5. Conclusion

In summary, we have confirmed that microglia are reduced in 30 day-old op/op mice compared to WT littermates but have normal levels of TH+ dopaminergic neurons. Unlike previous reports, we did not detect Aβ plaques in op/op mice up to 7 months of age. We detected a similar distribution of CD200 in the brains of op/op and WT controls, with lower levels of staining in the SN. Taken together these data demonstrate that op/op mice may be an important mouse model to study the role of glia in neurological diseases by breeding to transgenic mouse models of neurodegenerative diseases or pharmacological induced disease.

## Abbreviations

AD, Alzheimer's disease, APP, amyloid precursor protein, Aβ, amyloid β, CD, cluster differentiation, CNS, central nervous system, CSF, colony stimulating factor-1, MPTP, 1-methyl-4-1,2,3,6-tetrahydropyridine, op/op, osteopetrotic, PD, Parkinson's disease, SN, substantia nigra, TH, tyrosine hydroxylase, WT, wildtype,

## Authors' contributions

YK bred and harvested the tissue from the op/op mice, contributed to study design and edited the manuscript. CAL contributed to study design, data analysis and manuscript preparation. TJS performed the immunohistochemistry, image analysis, analyzed data, drafted the manuscript and obtained funding for these studies. All authors read and approved the final manuscript.
